# Perspectives of targeting LILRB1 in innate and adaptive immune checkpoint therapy of cancer

**DOI:** 10.3389/fimmu.2023.1240275

**Published:** 2023-09-13

**Authors:** Tobias Zeller, Ira A. Münnich, Roland Windisch, Patricia Hilger, Denis M. Schewe, Andreas Humpe, Christian Kellner

**Affiliations:** ^1^ Division of Transfusion Medicine, Cell Therapeutics and Haemostaseology, University Hospital, LMU Munich, Munich, Germany; ^2^ Medical Faculty, Otto-von-Guericke University Magdeburg, Magdeburg, Germany

**Keywords:** LILRB1 (ILT2), macrophages, cancer, phagocytosis, immune checkpoint blockade, antibody therapy, NK cells, T cells

## Abstract

Immune checkpoint blockade is a compelling approach in tumor immunotherapy. Blocking inhibitory pathways in T cells has demonstrated clinical efficacy in different types of cancer and may hold potential to also stimulate innate immune responses. A novel emerging potential target for immune checkpoint therapy is leukocyte immunoglobulin-like receptor subfamily B member 1 (LILRB1). LILRB1 belongs to the superfamily of leukocyte immunoglobulin-like receptors and exerts inhibitory functions. The receptor is expressed by a variety of immune cells including macrophages as well as certain cytotoxic lymphocytes and contributes to the regulation of different immune responses by interaction with classical as well as non-classical human leukocyte antigen (HLA) class I molecules. LILRB1 has gained increasing attention as it has been demonstrated to function as a phagocytosis checkpoint on macrophages by recognizing HLA class I, which represents a ‘Don’t Eat Me!’ signal that impairs phagocytic uptake of cancer cells, similar to CD47. The specific blockade of the HLA class I:LILRB1 axis may provide an option to promote phagocytosis by macrophages and also to enhance cytotoxic functions of T cells and natural killer (NK) cells. Currently, LILRB1 specific antibodies are in different stages of pre-clinical and clinical development. In this review, we introduce LILRB1 and highlight the features that make this immune checkpoint a promising target for cancer immunotherapy.

## Introduction

Immunotherapies are established as valid therapeutic options in the treatment of cancer (1). Various approaches have been evaluated pre-clinically and individual concepts have been translated into clinical application. Many novel approaches aim to unleash T cell responses in patients. Beside bispecific T cell engagers and chimeric antigen receptor (CAR) T cells, immune checkpoint inhibitors revealed therapeutic efficacy and their approval for clinical use opened up new avenues (2–4).

Immune checkpoint inhibitors are a class of therapeutic antibodies that block inhibitory interactions between receptors on immune cells and their ligands expressed on cancer cells (4). Immune checkpoint blockade in T cells using antibodies that target cytotoxic T-lymphocyte-associated protein 4 (CTLA-4), programmed cell death protein 1 (PD-1) or its ligand PD-L1, and more recently lymphocyte-activation gene 3 (LAG3) has proven therapeutic efficacy in different cancer types (4, 5). Yet, while in certain tumor entities considerable improvements were achieved, overall therapeutic response rates are still unsatisfactory (4). Beside T cells, also innate immune cells such as natural killer (NK) cells and macrophages exert a pivotal role in the recognition and elimination of malignant cells in the tumor microenvironment (6, 7). Thus, approaches that modulate innate immune cells are promising. Like T cells, innate immune cells are regulated by an interplay of activating and inhibitory receptors, which may serve as target antigens for therapeutic intervention. While currently the contribution of innate immune cells in CTLA-4 or PD-1 immune checkpoint therapies are under investigation (8), various antibodies or antibody-based fusion proteins targeting emerging immune checkpoints such as CD47, T cell immunoreceptor with Ig and ITIM domains (TIGIT) or natural killer group 2 member A (NKG2A) are developed and characterized for their effector functions (9–14).

In particular, approaches that orchestrate both innate and adaptive immune responses are of interest. Here, leukocyte immunoglobulin-like receptor subfamily B member 1 (LILRB1), also named immunoglobulin-like transcript (ILT) 2, monocyte/macrophage Ig-like receptor (MIR) 7 and CD85j, may represent an attractive target (9, 15, 16). This inhibitory receptor for human leukocyte antigen (HLA) class I is expressed by a variety of immune cells including certain cytotoxic lymphocytes and macrophages (17, 18). Thus, the engagement of this receptor was shown to impair cytotoxicity of LILRB1-expressing NK cells and CD8-positive T cells. More recently, HLA class I expression was demonstrated to protect cancer cells from phagocytosis by macrophages via interaction with LILRB1, rendering LILRB1 also a phagocytosis checkpoint (9, 19).

The therapeutic potential of antibodies targeting phagocytosis checkpoints has lately been highlighted by promising pre-clinical and clinical results obtained with antibodies targeting the ‘Don`t Eat Me!’ signal CD47 or its myeloid receptor signal regulatory protein (SIRP) α (9, 20, 21). Antibody blockade of either of them was shown to notably enhance antibody-dependent cellular phagocytosis (ADCP) by macrophages *in vitro* and in murine tumor xenograft models (20, 22–26). Clinically, encouraging results were achieved with the CD47 antibody magrolimab (hu5F9 G4) in combination with the CD20 antibody rituximab in a clinical phase Ib study in lymphoma patients and in combination with azacitidine and venetoclax in a phase I/II trial in acute myeloid leukemia (AML) patients, providing a rationale for further investigation of phagocytosis checkpoint inhibitors and their clinical development (27, 28).

Since interference with LILRB1 signaling may offer the opportunity to modulate various immune cell populations and to promote both innate and adaptive anti-tumoral immune responses, the LILRB1 immune checkpoint receives growing interest. Currently, the receptor is evaluated as a target pre-clinically and first clinical trials with individual LILRB1 targeted agents have been initiated to evaluate the potential of LILRB1 blockade in cancer patients. Here, the immune checkpoint LILRB1 is introduced putting a focus on its role in the regulation of monocytes, macrophages and cytotoxic lymphocytes, and perspectives for LILRB1 targeting in the treatment of cancer are outlined.

## LILRB1 genetics, structure and signaling

*LILRB1* is a member of the family of leukocyte inhibitory receptor (LIR) genes comprising eleven protein encoding members and two pseudo genes (18, 29–31). The genes are clustered within the leukocyte receptor complex (LRC) in proximity to genes of several related receptors such as killer cell immunoglobulin (Ig)-like receptors (KIR) and leukocyte-associated Ig-like receptors on human chromosome 19q13.4. LIR are grouped into two subfamilies (29, 32, 33). Subfamily A consists of five cell surface receptors (LILRA1, LILRA2, LILRA4-6) that exert activating functions by association with the immunoreceptor tyrosine-based activation motif (ITAM) containing fragment crystallizable (Fc) region receptor (FcR) γ chain and the soluble member LILRA3. The subfamily B comprises five inhibitory receptors (LILRB1-5), which are type I transmembrane proteins characterized by two to four extracellular Ig-like domains for ligand binding and two to four intracellular immunoreceptor tyrosine-based inhibitory motifs (ITIM) for signal transduction (18, 32, 34). LILRB are specific to primates and humans, but orthologs exist in other species such as paired immunoglobulin-like receptor B (PirB) 18 and gp49B1 in mice (31, 34). LILRB1 together with LILRA2 (ILT1) were the first members of the LIR receptor family identified in 1997 by Samaridis and Colonna (35). In the same year, LILRB1 was shown to be a receptor for cellular HLA class I and for the human cytomegalovirus (CMV) UL18 gene product, a viral homolog of HLA class I (17, 36, 37).

**Figure 1:**
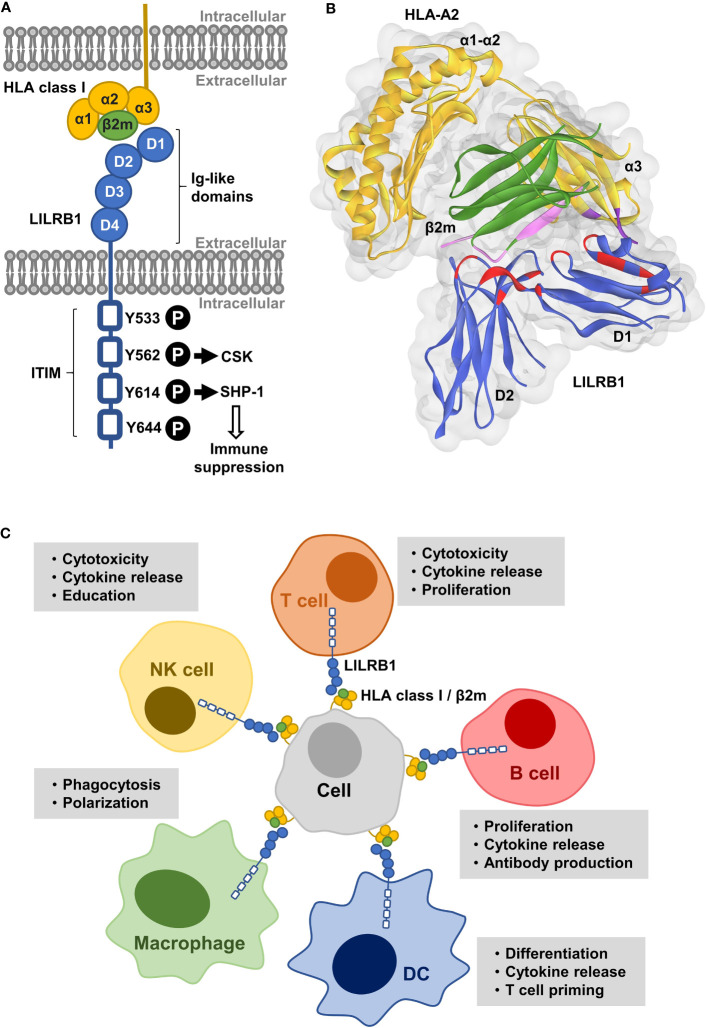
Structure of LILRB1 and immune inhibitory functions. **(A)** LILRB1 contains four extracellular immunoglobulin (Ig)-like domains (D1-D4). The binding site for HLA class I/β2-microglobulin (β2m) molecules localizes to the apical D1-D2 region. The intracellular portion comprises four immunoreceptor tyrosine-based inhibitory motifs (ITIM). Critical phosphorylation tyrosine residues involved in the recruitment of SHP-1 and CSK within ITIM domains are indicated. **(B)** Illustration of the LILRB1 D1-D2 domains (blue) bound to HLA-A2/β2m (depicted in yellow and green, respectively) according to the previously published crystal structure of the complex (38). The LILRB1 D1 domain mainly interacts with the HLA class I α3 domain and the D1-D2 interdomain hinge region with the conserved β2m subunit. The contact sites in LILRB1, HLA-A2 and β2m are highlighted in red, pink and purple, respectively. The ribbon drawing was generated using the pdb file 1P7Q as a template and Discovery Studio Visualizer software (Dassault Systèmes Biovia Corp). **(C)** LILRB1 is expressed by various immune cell populations including subpopulations of T cells and natural killer (NK) cells, B cells, macrophages and dendritic cells (DC), and contributes to the regulation of diverse immune cell functions.

LILRB1 was identified as a 110-120 kDa, 651 amino acid glycoprotein, which is composed of four Ig-like domains in its extracellular part and a cytoplasmic tail of 167 amino acids containing four ITIM or ITIM-like sequences (**Figure 1**) (36). LILRB1 exists in 13 different isoforms due to alternative splicing. Like other LIR, LILRB1 is expressed also as a soluble isoform, which is capable of ligand binding and may interfere with interactions between ligand and cell surface LILRB1 (39). As related KIR, LILRB family members are polymorphic, although to a lesser extent, with LILRB1 showing a considerable allelic diversity (33, 40, 41).

LILRB1 functions as an inhibitory receptor by suppressing the activity of intracellular kinases. Upon LILRB1 ligation, tyrosines within the ITIM are phosphorylated and the phosphatase SRC-homology 2 domain-containing phosphatase (SHP) 1 is recruited (17, 36, 42, 43). The tyrosine residues Y562, Y614 and Y644 were shown to be involved in SHP-1 recruitment with Y614 being the main docking site, while tyrosine phosphorylation of the receptor involved Y533 (44, 45). In addition, recruitment of SHP-2 has been reported (46). The activated phosphatases dephosphorylate ITAM and suppress key kinases involved in the activation of immune cells including spleen tyrosine kinase (SYK), SRC, zeta-chain associated protein kinase 70-kDa, phosphatidylinositol-4-phosphate 3-kinase and others (43). LILRB1 also interacts with the C-terminal SRC kinase (CSK), an important negative regulator of SRC kinases, which preferentially binds to the ITIM containing Y562 (45). In certain situations, LILRB1 was demonstrated to exert stimulatory function (34, 47). The underlying mechanistic events have not been unraveled yet, but as an explanation, the C-terminal ITIM was suggested to function as an immunotyrosine-based switch (ITSM) domain, which converts inhibitory into activating signaling.

## Broad recognition of HLA class I by LILRB1

In contrast to KIR, which are restricted to the recognition of distinct HLA class I allele variants, LILRB1 is a receptor for a wide spectrum of HLA class I molecules. Thus, LILRB1 not only interacts broadly with allelic variants of classical HLA-A, HLA-B and HLA-C antigens but also binds the non-classical HLA-E, HLA-F and HLA-G molecules (38, 48–52). Yet, affinities vary, and equilibrium binding constants are between 2 and 100 µM (51–54). HLA-F and HLA-G, the latter of which plays a crucial role in tumor immune escape owing to its immune suppressive function, were suggested to bind with a particular high affinity. Importantly, LILRB1 does not recognize β2-microglobulin (β2m)-free forms of HLA molecules.

The binding site for HLA class I localizes to the apical LILRB1 D1-D2 region, which contains two Ig-like domains (**Figure 1A**) (38, 52, 55). Crystal structure analysis showed that the D3-D4 domains are not involved (56, 57). Binding of HLA molecules may occur both *in cis* and *in trans*, due to a highly flexible interdomain hinge region (55, 57, 58). The observed broad specificity for HLA class I molecules results from the distinct binding sites within the HLA complex. As revealed by crystallographic studies, LILRB1 binds the comparably low polymorphic α3 domain of HLA via the tip of its D1 domain and the conserved β2-microglobulin (β2m) subunit mainly via the D1-D2 interdomain hinge region (**Figure 1B**) (38, 52, 55). Yet, LILRB1 polymorphisms affect HLA class I binding and allelic variants differing in their D1-D2 region exert different affinities (40). In addition, allelic variation in the α3 domains of HLA class I molecules influences binding affinity (48). Of note, also other members of the LILR family (i.e. LILRB2, LILRB5, LILRA1-3) bind HLA class I molecules, but differ in terms of β2m dependency (59). For example, LILRB2 binds in a β2m-independent mode and also recognizes open conformers lacking β2m due to a few differences in its amino acid sequence resulting in a predominant recognition of the α3 domain (48, 60).

As other members of the LILRB subfamily LILRB1 has multiple ligands (18, 34). Thus, LILRB1 also binds the two calcium-binding cellular proteins S100A8 and S100A9 (61) and interacts with pathogen-derived ligands. These include the above-mentioned UL18 (36, 62), dengue virus and bacterial antigens (29, 63) as well as certain *Plasmodium falciparum* repetitive interspersed families of polypeptides (RIFINs) (64).

## LILRB1 in the regulation of immune cells

LILRB1 is the most widely expressed member of the LILRB subfamily and is found in various immune cell populations including monocytes, macrophages, dendritic cells (DC), granulocytes, mast cell progenitors, osteoclast precursors as well as B cells and subpopulations of T cells and NK cells (17, 58, 65–68). LILRB1 is involved in the regulation of diverse processes including immune cell differentiation and proliferation, cytokine release, antigen presentation, phagocytosis, cytotoxicity as well as antibody production (**Figure 1C**) (17, 18, 69–72). Like other members of the LIR family, LILRB1 plays a role in different diseases including pathogen infection, certain autoimmune diseases and cancer (18, 29, 34, 59, 73–75). Particularly its expression by monocytes/macrophages and cytotoxic lymphocytes, which represent relevant effector cell populations in current immunotherapeutic approaches, renders this receptor an attractive target for cancer immunotherapy.

### Monocytes/macrophages

Monocytes and macrophages express all members of the LILRB subfamily (32) with LILRB1 being uniformly expressed in monocytes as well as in *ex vivo* differentiated macrophages (17, 19, 76). LILRB1 cross-linking reduced Ca^2+^ mobilization upon HLA-DR activation (17), and importantly, interfered with activation through stimulation of FcγR (42). Thus, co-ligation of LILRB1 and the activating FcγRI inhibited protein tyrosine kinases and led to reduced tyrosine phosphorylation of the FcRγ-chain and SYK, resulting in decreased intracellular Ca^2+^ mobilization. Therefore, LILRB1 activation was suggested to impair FcγR-mediated effector functions of therapeutic antibodies such as ADCP. Indeed, LILRB1 plays an important role in the regulation of phagocytosis by macrophages (19). The function of LILRB1 as a phagocytosis checkpoint was identified in an attempt to unravel inhibitory pathways that impair the pro-phagocytic activity of the CD47 antibody magrolimab using a panel of solid tumor cell lines (19). *LILRB1* gene knockout experiments showed that LILRB1 represents the major receptor for HLA molecules in the regulation of phagocytosis by macrophages.

LILRB1 may also be involved in the regulation of macrophage polarization. Macrophages exist in a range of activation states between the two extremes of classically activated, pro-inflammatory M1 and alternatively activated M2 macrophages (77, 78). A regulatory role was suggested for LILRB1, since knockout of the *LILRB1* gene in macrophages *in vitro* resulted in a higher proportion of macrophages displaying an M1 immunophenotype, possibly by abrogation of interactions of LILRB1 with HLA molecules *in cis* (19). This function has already been demonstrated for LILRB2. LILRB2 antagonism has been reported to drive tumor-infiltrating myeloid cell polarization toward an inflammatory, M1-like phenotype and to enhance pro-inflammatory and adaptive immune responses (79).

### Dendritic cells

DC differentiated *ex vivo* from monocytes (moDC) or CD34-positive progenitor cells strongly express LILRB1 (17, 69, 70, 80). During differentiation, LILRB1 was shown to be upregulated and expression was preserved upon DC maturation (70). LILRB1 is also expressed by most human dendritic subsets from the peripheral blood (65). Thus, LILRB1 was found on type 2 conventional DC, plasmacytoid DC and 6-sulfo-LacNAc-positive DC. In these DC subtypes, LILRB1 is upregulated upon toll-like receptor (TLR) stimulation with lipopolysaccharide (LPS) or imiquimod. In contrast, type 1 conventional DC lack LILRB1 expression, even after exposure to TLR ligands. LILRB1 has been suggested to modulate the differentiation of DC and to regulate their functions (70). Thus, DC differentiated from monocytes in the presence of a LILRB1 ligand remained CD14 expression and exerted low HLA-DR levels. The cells did not respond to LPS-induced maturation, did not secrete characteristic cytokines such as interleukin (IL)-10, IL-12 or TGFβ, and exerted a weak T cell stimulating activity (70, 81). Moreover, LILRB1 engagement was shown to inhibit activation by stimulation of the osteoclast-associated receptor (OSCAR), an activating FcR γ- chain associated myeloid receptor involved in antigen presentation (69). Thus, co-ligation of LILRB1 and OSCAR resulted in reduced Ca^2+^ mobilization, impaired cytokine secretion, and a diminished potency to stimulate the proliferation of antigen-specific T cells *in vitro* (69).

### NK cells

In contrast to monocytes, only a subpopulation of NK cells displays LILRB1 and at lower levels (17). Cell type specific divergent expression was attributed to different promotors in monocytes and NK cells (82). In healthy conditions, LILRB1 expression varies considerably between individuals. The receptor was found on the cell surface of 23% to 77% of NK cells (17), and the observed variation between individuals was linked to polymorphisms in the *LILRB1* gene (83). In general, LILRB1 expression differs in NK cell subpopulations and is higher in CD56^dim^ NK cells, which express FcγRIIIA and are predominant in the peripheral blood, than in CD56^bright^ NK cells, which lack FcγRIIIA expression (84). In particular, LILRB1 is expressed by terminally differentiated NK cells expressing CD57 and numerous KIR and by virus-induced adaptive NK cells (85–88). Recent findings show that at least under certain conditions LILRB1 contributes to NK cell education - a process that counterbalances the responsiveness of individual NK cells with their sensitivity for inhibition by cognate HLA class I molecules (89, 90). Importantly, LILRB1 engagement by HLA-G impairs the formation of the NK cell activating synapse by inhibiting the polarization of lytic granules and the microtubule organizing centre and impaired filamentous actin accumulation (91). Upon ligation, LILRB1 inhibits Ca^2+^ mobilization, the release of inflammatory cytokines such as interferon (IFN) γ and impairs both natural cytotoxicity and ADCC (17, 91–96).

### T cells

LILRB1 is also displayed by certain T cells, yet a considerable variation in the frequency of T cells expressing cell surface LILRB1 exists between individuals (97). In healthy donors, LILRB1 is found on the cell surface of about 20% of αβ T cells in younger individuals, but this can increase up to over 50% with age or upon chronic infections (98, 99). LILRB1 surface expression is found predominantly in CD8-positive T cells, in contrast to CD4-positive T cells, which rarely display LILRB1 (98, 100). LILRB1-positive T cells revealed a reduced potential to proliferate but were capable of producing IFN-γ upon T cell receptor (TCR) stimulation (101). Further expression analysis revealed that in CD8-positive T cells LILRB1 was preferentially displayed by the terminally differentiated population of effector memory T cells re-expressing CD45RA (T_EMRA_) and marks a T cell population with strong effector functions and a high content of intracytoplasmic perforin (99, 101–103). In addition, cell surface LILRB1 was found also on a subset of effector memory T cells (T_EM_). Interestingly, LILRB1 and PD-1 are expressed on distinct T cell populations and induced expression of PD-1 by TCR stimulation was found preferentially in LILRB1-negative T cells *in vitro* (102).

In experiments with superantigen pulsed antigen presenting cells (APC) LILRB1 was found to colocalize with the T cell receptor (TCR) at the immunological synapse (104). Engagement of LILRB1 interfered with TCR/CD3 signaling and impaired actin cytoskeleton reorganization, cytotoxicity, cytokine production, and attenuated proliferation (17, 93, 94, 104–106). Moreover, LILRB1 was shown to compete with CD8 for binding to HLA class I molecules (53). Thus, in addition to impairing T cell responses by ITIM signaling, LILRB1 may interfere with T cell activation by blocking the binding of CD8. T cells can also acquire LILRB1 from monocytes by trogocytosis (107). The acquired LILRB1 molecules had signaling properties and were shown to regulate T cell functions. LILRB1 is also expressed by subsets of γδ T cells and contributes to their regulation (17, 108). For example, gene knock-down of β2m or antibody blockade of LILRB1 enhanced lysis of lymphoma cells by γδ T cell clones (108).

### B cells

LILRB1 expression in B cells increases throughout maturation in the bone marrow and is maintained at the plasma cell stage (16, 82). In peripheral blood, mature B cell populations including naïve and memory B cells homogenously express LILRB1, whereas only a fraction of transitional B cells were stained positive for LILRB1 (82). LILRB1 has been suggested to participate in the regulation of B cell functions. Thus, antibody-mediated crosslinking of LILRB1 interfered with B cell receptor activation resulting in reduced Ca^2+^ mobilization (17). Subsequent findings indicated that LILRB1 activation dampened IgG production, inhibited isotype switching and impaired cytokine production (71). In addition, HLA-G aggregated on nanoparticles interfered with B cell responses by binding to LILRB1 (72). In these experiments, inhibition of T cell-dependent as well as T cell-independent B cell responses was observed and aggregated HLA-G reduced B cell proliferation, antibody secretion and cytokine release.

## The LILRB1-HLA axis in cancer

Increasing evidence indicates that LILRB1 like other members of the LILRB family plays a role in cancer development, treatment and tumor immunotherapies (43). In the tumor microenvironment, LILRB1 is expressed by different immune cells and was suggested to support tumor growth indirectly by contributing to the repression of multiple anti-tumoral functions. LILRB1-mediated immune suppression relies on the expression of HLA class I molecules and their expression levels. Interestingly, also malignant cells from certain tumor entities, in particular lymphomas and leukemias, express LILRB1.

### Modulation of anti-tumoral immune responses by LILRB1 and HLA class I

Alterations in HLA expression that emerge during the evolution of immune escape variants are frequently found in tumors (109, 110). Down-modulation of classical HLA molecules enables tumor cells to evade from attack by T cells, which typically recognize peptides presented on HLA molecules. In contrast, HLA class I molecules function as markers of self and inhibit NK cell functions (11). As a consequence, reduced or loss of HLA class I expression renders tumor cells susceptible to NK cell cytotoxicity. Typically, HLA class I recognition involves KIR and NKG2A receptors, but also LILRB1 plays a role. Thus, masking of LILRB1 enhanced NK cell cytotoxicity against HLA class I-expressing leukemia cells, in particular when KIR or NKG2A were blocked simultaneously (111). LILRB1 was suggested to interfere with activation of NK cells upon engagement of natural killer group 2 member D (NKG2D), an important stimulatory NK cell receptor that regulates natural cytotoxicity, and to inhibit lysis of leukemic cells with ectopic expression of the NKG2D ligand MHC class I chain-related protein A (MIC A) (92).

Intriguingly, HLA class I molecules not only inhibit NK cell-mediated lysis of tumor cells but also function as ‘Don`t Eat Me!’ signals that prevent malignant cells from phagocytic uptake by macrophages through LILRB1 engagement (19). In the tumor microenvironment, tumor-associated macrophages (TAM) contribute to cancer progression by supporting cancer cell survival and proliferation, promotion of angiogenesis, and suppression of immune responses (77, 78, 112). Yet, macrophages are also able to eliminate malignant cells by phagocytosis and to promote anti-tumoral functions of lymphocytes. The role of MHC class I in the regulation of anti-tumoral responses by macrophages was demonstrated in xenograft studies using genetically modified human tumor cells expressing a mouse human chimeric β2m protein to confer interaction with murine macrophages (19). These experiments revealed that MHC class I protected tumor cells from macrophage attack *in vivo*. Genetic deletion of HLA class I cell surface molecules augmented ADCP by anti-epithelial cell adhesion molecule (EpCAM) or anti-epidermal growth factor receptor (EGFR) antibodies, suggesting that the HLA class I:LILRB1 axis may compromise the therapeutic efficacy of tumor targeting antibodies. Thus, loss of HLA class I may sensitize tumor cells to phagocytosis.

Tumors may also impair immune cells by neo-expression of non-classical HLA molecules such as HLA-G. HLA-G expression was described in various tumors including gastric, colorectal, lung, breast, hepatocellular and pancreatic cancer as well as chronic lymphocytic leukemia and was associated with tumor progression and a worse outcome (113–116). Both cell membrane-bound HLA-G and its soluble form exert pleiotropic immune suppressive functions on various immune cells including T cells, NK cells and APC, which involve LILRB1 signaling (114). For instance, in NK cells, interaction of HLA-G and LILRB1 was shown to counteract signaling and induction of cytotoxicity by interaction of the activating NK cell receptor NKG2D with its ligand MIC A on tumor cells (117). Interestingly, HLA-G upregulates the expression of LILRB1 in NK cells, T cells and APC *in vitro* (118), which may further potentiate immune suppression.

### Expression of LILRB1 in the tumor microenvironment

The analysis of cells in the tumor microenvironment of different types of solid tumors confirmed that LILRB1 was mainly expressed in the tumor stroma with TAM representing the major LILRB1 expressing immune cell population (113). Of note, LILRB1 was found to be displayed by TAM from different types of solid tumors including colon carcinoma, head and neck cancer, non–small cell lung cancer, renal cell carcinoma and by lymphoma associated macrophages (19, 76, 113). The analysis of the tumor microenvironment in gastric cancer revealed that LILRB1-positive TAM had an M2-like phenotype. Their numbers correlated with the levels of immune suppressing cytokines and were associated with T cell exhaustion and increased expression of inhibitory immune checkpoints such as PD-1 and CTLA-4 (119). Beside TAM, LILRB1 was expressed by a significant number of NK cells (113). In certain tumors, the fraction of LILRB1 expressing NK cells was increased in the tumor microenvironment. For example, in prostate cancer, a higher percentage of tumor infiltrating NK cells expressed LILRB1 in comparison to NK cells from control tissues (120). Tumor-induced upregulation of LILRB1 was supported further by subsequent findings revealing that certain tumor cells, i.e. prostate cancer cells and glioblastoma cells, are capable of inducing the expression of LILRB1 on NK cells *in vitro* (120, 121). In addition, cell-to-cell contact with M2 polarized macrophages augmented LILRB1 expression in NK cells, which led to reduced cytotoxicity and cytokine production (122). In contrast, LILRB1 was more rarely expressed in tumor infiltrating T cells and rather restricted to CD8-positive T cells with a T_EMRA_ phenotype. Of note, in the peripheral blood of cancer patients an increased frequency of LILRB1 expressing NK and T cells was observed compared to healthy controls (92, 96, 113, 120, 123, 124).

The expression of LILRB1 in the tumor microenvironment may have prognostic features. In gastric cancer for example, elevated LILRB1 expression was associated with advanced tumor stages, increased risk of recurrence and inferior survival (119). In prostate cancer, enhanced levels of LILRB1 mRNA were associated with a shorter biochemical recurrence-free survival according to prostate specific antigen levels in the blood (125). Moreover, in ovarian cancer a high content of LILRB1-positive immune cells was correlated with shorter survival, and worse adjuvant chemotherapy responses (126).

LILRB1 is also expressed by malignant cells of different tumor types (43). Thus, LILRB1 expression was reported in AML (127), certain T cell lymphomas (128–130), B-lineage lymphomas and leukemias (131–133), gastric cancer (134), ovarian cancer (126), and renal cell carcinoma (135). LILRB1 expression by tumor cells may have different consequences. For example, LILRB1 expression protected cutaneous T-cell lymphoma cells from CD3/TCR activation induced cell death (128), while in malignant B cells LILRB1 engagement dampened tumor cell proliferation and induced cell cycle blockade (132). Intriguingly, the expression of LILRB1 may increase the susceptibility of cancer cells to an attack by immune cells. In multiple myeloma, loss of LILRB1 has been suggested to contribute to immune escape by reducing their susceptibility to NK cell mediated lysis (133). The LILRB1 ligand S100A9 expressed by NK cells was suggested to play a role and effects of LILRB1 expression on cytotoxicity were diminished by S100A9 blockade. Moreover, LILRB1 expressed by lymphoma cells sensitized these cells to lysis by certain γδ T cells, presumably by co-stimulation of the effector cells through ligation of HLA class I on γδ T cells (131).

## Antibody immune checkpoint blockade of LILRB1

**Figure 2:**
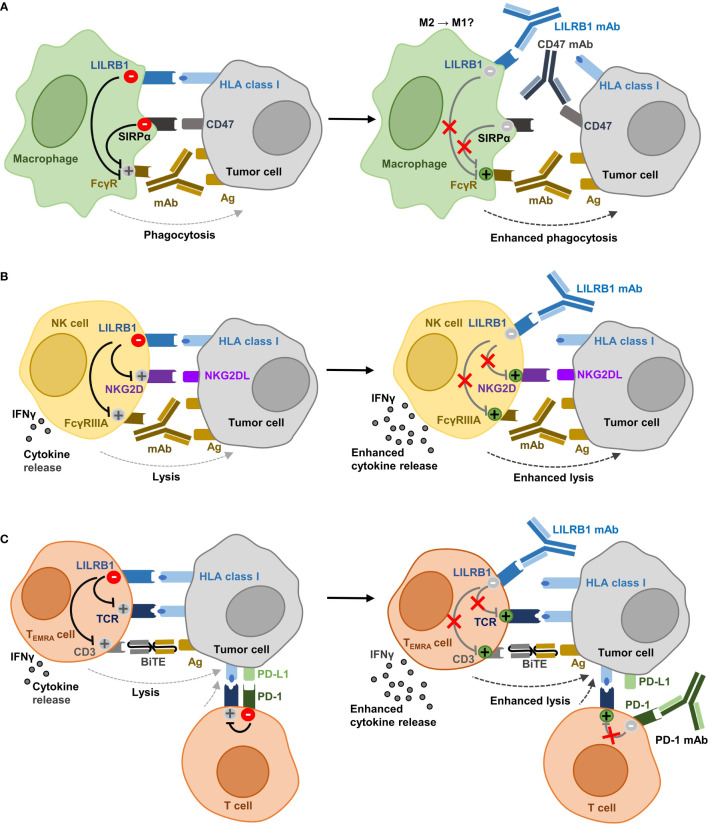
Potential effector functions of anti-LILRB1 antibodies in cancer immunotherapy. **(A)** Recognizing HLA class I molecules, LILRB1 functions as an immune checkpoint in macrophages and inhibits together with the CD47 receptor SIRPα phagocytosis of tumor cells. Antagonistic LILRB1 antibodies enhance phagocytosis and promote ADCP by therapeutic antibodies that target an antigen expressed by the tumor and which provide an ‘Eat Me!’ signal by ligation of activating FcγR. LILRB1 blockade also cooperates with CD47 antibodies in enhancing ADCP. Since a role for LILRB1 has been implicated in macrophage polarization, the blockade of the HLA class I:LILRB1 axis may support re-programming of TAM from an M2-like phenotype towards M1. **(B)** In NK cells, LILRB1 engagement impairs both cytokine release and cytotoxicity. LILRB1 ligation hampers lysis of cancer cells induced by NK cell lysis receptors such as NKG2D or by activation of FcγRIIIA through therapeutic antibodies targeting a tumor-expressed antigen. LILRB1 antibody blockade enhances cytokine release, natural cytotoxicity and ADCC. **(C)** In CD8-positive T cells, mainly T_EMRA_ cells, LILRB1 inhibits cytokine release and cytotoxic functions triggered by activation of the T cell receptor. LILRB1 ligation also impairs tumor cell lysis induced by therapeutic BiTE molecules specific for a tumor cell expressed antigen and activating CD3. Antagonistic LILRB1 antibodies promote cytokine release and T cell cytotoxicity and may be employed to enhance the efficacy BiTE molecules. The expression of PD-1 and LILRB1 on different T cell subsets renders LILRB1 blockade attractive for combination with anti-PD-1 antibodies to abrogate inhibitory signaling in both T cell populations (Ag, antigen; NKG2D, natural killer group 2 member D; NKG2DL, NKG2D ligand; FcγRIIIA, Fcγ receptor IIIA; TCR, T cell receptor; BiTE, bispecific T cell engager; PD-1, programmed cell death 1; PD-L1, PD-1 ligand 1).

The important inhibitory function of the HLA class I:LILRB1 axis on phagocytosis and its role in the regulation of NK cells and T cells may offer the opportunity for therapeutic intervention using immune checkpoint inhibitors (9). LILRB1 antagonism may diminish inhibitory signaling and shift the balance towards activating signaling in immune cells – thereby promoting phagocytosis and/or cellular cytotoxicity. In combination with other therapeutic monoclonal or bispecific antibodies targeting tumor cells such inhibitors may enhance the efficacy of antibody therapy by enhancing key functions such as ADCP or ADCC (**Figure 2**).

Clinically applied immune checkpoint inhibitors are often IgG4 antibodies, which exert lower affinity to FcγR than IgG1, the most commonly used isotype for therapeutic antibodies. Fc engineered, Fc-silent versions of IgG antibodies, in which both FcγR and complement binding is abolished by introduction of distinct amino acid substitutions, may represent an alternative (136, 137). Such engineered antibodies may prevent undesired binding to various FcγR expressed by different immune cell populations and abrogate Fc-dependent elimination of LILRB1 expressing effector cell populations by ADCC, ADCP or complement dependent cytotoxicity. The pronounced homology between LILRB1 and LILRB2, which is more restricted to the myeloid lineage in its expression, allowed the generation of antibodies that bind both receptors (138). Such dual antagonist strategies may be beneficial in respect of the HLA specificity and inhibitory functions of both receptors in myeloid cells. In addition to monoclonal antibodies, receptor-ligand interactions can also be abrogated using recombinant fusion proteins between a cognate ligand and the human Fc domain (139, 140). Corresponding HLA-β2m-Fc fusion proteins may also offer the opportunity to block other HLA class I receptors, including LILRB2, concomitantly (141).

### LILRB1 blockade in macrophages

In initial experiments, Barkal and colleagues employed the murine hybridoma antibody clone GHI/75 to disrupt the HLA class I:LILRB1 axis (19). Similar to HLA class I-specific fragments antigen binding (Fab), this anti-LILRB1 antibody enhanced the phagocytosis of HLA class I-expressing solid tumor cell lines *in vitro*, but required combination with the CD47 directed IgG4 antibody magrolimab to become effective (19). Notably, CD47 IgG4 antibodies may exert dual function by blocking CD47 at the tumor cell site and engaging activating FcγR at the effector cell side to provide an ‘Eat Me!’ signal (9, 19, 142). Regarding the suggested interplay between the two axes CD47:SIRPα and HLA class I:LILRB1 in counteracting effective ADCP of tumor cells as demonstrated for anti-EpCAM or anti-EGFR antibodies (19), additional antibody combinations were studied. For example, a chimeric, Fc-silent variant of antibody GHI/75 with abrogated FcγR binding was generated, to prevent potential occurring effects by binding of this antibody to FcγR (76). In combination with a CD20 antibody (i.e. rituximab or obinutuzumab) for FcγR engagement and an Fc-silent CD47 antibody to inhibit SIRPα signaling, LILRB1 antibody blockade further enhanced the phagocytic uptake of lymphoma cells by macrophages *in vitro* compared to the combination of CD20 antibodies and CD47 blockade only. This was observed in studies with non-polarized macrophages as well as macrophages that were polarized towards M1 or M2 phenotypes *ex vivo*, and lymphoma-associated macrophages isolated from the bone marrow of lymphoma patients. The blockade of LILRB1 alone however was not sufficient to induce phagocytosis in this study. In addition, the LILRB1 antibody was not effective when combined only with either a CD20 or a CD47 antibody. These results suggested that both FcγR engagement by the tumor targeting antibody and blockade of the CD47:SIRPα axis were required (76). In contrast, characterization of the humanized IgG4 anti-LILRB1 antibody BND-22 revealed that this antibody was effective also as single agent in inducing phagocytosis of tumor cells by human macrophages both *in vitro* and in a xenograft *in vivo* model in which both human macrophages and cancer cells were transferred (113). In addition, the antibody enhanced ADCP by the anti-EGFR antibody cetuximab *in vitro*, thereby providing further evidence that LILRB1 blockade may enhance the therapeutic efficacy of tumor targeting antibodies by enhancing their ADCP function.

Regarding the tumor promoting and immune suppressive functions of TAM, reprogramming TAM to a pro-inflammatory state may inhibit tumor growth. In fact, LILRB2 antagonistic antibodies were shown to exert this immunomodulatory function (79). Yet, whether LILRB1 blockade allows promoting polarization towards an inflammatory M1-like phenotype and whether this may offer the opportunity to also reprogram TAM, has not been addressed experimentally to our knowledge and will require further investigation (**Figure 2A**).

### LILRB1 blockade in NK cells

NK cells play an important role in immunosurveillance against cancer due to their natural cytotoxic functions and represent an important effector cell population for therapeutic antibodies as mediators of ADCC. LILRB1 blockade may allow both promoting natural cytotoxicity and enhancing ADCC (**Figure 2B**). For example, antibody masking of LILRB1 was demonstrated to promote the activation of NK cells from chronic lymphocytic leukemia (CLL) patients, to enhance NK cell proliferation when combined with the immunomodulatory drug lenalidomide and to promote lysis of CLL cells (96). Similarly, enhanced NK cell cytotoxicity against glioblastoma cells was observed *in vitro* when LILRB1 was blocked (121). A humanized, Fc-engineered anti-LILRB1 IgG antibody, B1-176, with reduced FcγR binding was shown to disrupt LILRB1 signaling in co-culture experiments with LILRB1 reporter cells and various tumor cell lines (92). The antibody enhanced natural cytotoxicity by the NK cell line NKL against HLA class I-positive leukemia cells and particularly supported lysis induced by NKG2D engagement. In addition, antibody B1-176 promoted anti-tumoral activities of NK cells in ALL or multiple myeloma xenograft experiments *in vivo*. Regarding ADCC, LILRB1 was shown to hamper cetuximab-mediated ADCC by NK cells. Thus, antagonistic LILRB1 antibodies restored the cytotoxic activity of NK cells from triple-negative breast cancer patients and enhanced ADCC (123).

### LILRB1 blockade in T cells

T cells are an attractive effector cell population in cancer immunotherapy, in which T cell checkpoint inhibitors or bispecific T cell engager (BiTE) molecules have shown promising results (3, 4). The ability of anti-LILRB1 antibodies to enhance T cell activation was demonstrated in mixed lymphocyte reactions with allogeneic T cells and DC, in which the antibody BND-22 enhanced T cell activation as verified by IFN-γ release (113). Recent findings suggest that LILRB1 engagement may impair the efficacy of both BiTE and PD-1 immune checkpoint inhibitors (**Figure 2C**). LILRB1-expressing T_EMRA_ cells represent an important T cell population for CD3 bispecific antibodies with a high cytotoxicity (102). The anti-LILRB1 antibody GHI/75 has shown potential to enhance T cell cytotoxic activity mediated by an [anti-MART-1 × CD3] BiTE molecule by disrupting the interaction of LILRB1 with HLA-G expressed by malignant melanoma cells (102). Regarding PD-1 immune checkpoint blockade, LILRB1 upregulation has been discussed to be a potential mechanism of resistance. Thus, increased expression of LILRB1 was reported in nearly half of malignant melanoma patients after treatment with the PD-1 directed antibody nivolumab (113). Interestingly, combining anti-PD-1 and anti-LILRB1 antibodies synergistically enhanced the secretion of TNF-α by autologous peripheral blood mononuclear cells co-incubated with colon cancer cells *in vitro* and improved therapeutic effects were observed for the antibody combination *in vivo* (113). Moreover, since PD-1 and LILRB1 are described to be expressed by different T cell subsets, co-blockade of both receptors may provide an opportunity to abrogate inhibitory signaling pathways in both subsets (102).

## LILRB1 targeting antibodies or immunoconstructs in clinical development

The promising pre-clinical results achieved by targeting LILRB1 have paved the way for first clinical trials with individual LILRB1 antagonists in cancer patients (**Table 1**). The anti-LILRB1 antibody BND-22/SAR444881 (Biond Biologics/Sanofi) is a humanized monoclonal antibody of the IgG4 isotype (113). The antibody was shown to bind LILRB1 selectively and not to cross react with other members of the LILR family. By binding the LILRB1 D1-D2 domains, the antibody prevented engagement of LILRB1 by both classical HLA class I and HLA-G molecules. *In vitro*, BND-22/SAR444881 enhanced NK cell-mediated tumor cell lysis, T cell activation and phagocytosis. BND-22 improved the therapeutic efficacy of anti-EGFR or anti-PD-1 antibodies in murine tumor models. The antibody is currently evaluated in an ongoing phase 1/2 clinical trial (clinical trials.gov identifier: NCT04717375) in patients with advanced solid tumors. BND-22/SAR444881 is applied alone or in combination with either the anti-EGFR antibody cetuximab or the anti-PD-1 antibody pembrolizumab.

**Table 1:** Current clinical studies with LILRB1 targeting antibodies or fusion proteins.

Antibody/immune construct (company)	Format	Target	Disease	Intervention	Phase	Clinical trials. gov identifier
BND-22/SAR444881 (Biond Biologics/Sanofi)	humanized IgG4	LILRB1	advanced solid tumors	BND-22/SAR444881 alone BND-22/SAR444881 + anti-PD-1 (pembrolizumab) BND-22/SAR444881 + anti-EGFR (cetuximab)	I/II	NCT04717375
NGM707 (NGM Biopharmaceuticals)	humanized IgG	LILRB1, LILRB2^1^	advanced solid tumors	NGM707 alone NGM707 + anti-PD-1 (pembrolizumab)	I/II	NCT04913337
AGEN1571 (Agenus)	human IgG4κ	LILRB1, LILRB2^2^	advanced solid tumors	AGEN1571 alone AGEN1571 + anti-PD-1 (balstilimab) AGEN1571 + anti-CTLA-4 (botensilimab)	I	NCT05377528
IOS-1002 (ImmunOs Therapeutics)	Human HLA-β2m-Fc fusion	LILRB1, LILRB2, KIR3DL1	advanced solid tumors	IOS-1002 alone IOS-1002 + anti-PD-1	I	NCT05763004

^1^Weak cross reactivity with LILRB2, ^2^dual specificity for LILRB1/2.

AGEN1571 (Agenus Inc.) is a fully human IgG4κ monoclonal antibody (143). AGEN1571 demonstrated weak cross reactivity to LILRB2, but did not bind other LILR. The antibody was shown to promote M1 polarization and enhanced cytokine release and activation of NK and CD8-positive T cells alone and in combination with a PD-1 blocking antibody *in vitro*. AGEN1571 is currently tested in a phase I clinical trial (NCT05377528) in patients with advanced solid tumors. AGEN1571 is evaluated as a single agent or in combination with either the PD-1 inhibitor balstilimab and/or the anti-CTLA-4 antibody botensilimab.

NGM707 (NGM Biopharmaceuticals, Inc.) is a humanized IgG antibody with dual specificity for both LILRB1 and LILRB2 (138). The antibody exerts dual antagonistic functions by blocking the interaction of both receptors with classical HLA class I as well as HLA-G molecules. NGM707 promoted phagocytosis of cancer cells by macrophages and increased cytotoxicity by NK and CD8-positive T cells. In combination with pembrolizumab, NGM707 enhanced activation of T cells by macrophages in mixed lymphocyte reactions. NGM707 is currently tested as monotherapy or in combination with pembrolizumab in advanced or metastatic solid tumor malignancies in a phase 1/2 trial (NCT04913337). First results have been reported for the NGM707 monotherapy dose escalation, in which the antibody was well tolerated up to a dose level of 1800 mg, and a maximum tolerated dose was not reached. NGM707 monotherapy demonstrated early signs of efficacy with the best overall responses of stable disease in six patients and non-complete response/non-progressive disease in one patient of 20 response-evaluable patients. Interestingly, signs of myeloid reprogramming in form of reduced CD163 expression were observed post-treatment (144).

IOS-1002 (iosH2, ImmunOs Therapeutics AG) is an optimized fusion protein consisting of an peptide-free engineered variant of HLA-B*57 (amino acid substitutions: A46E/V97R) fused to human β2m and the human IgG4 Fc domain (141). The amino acid substitutions were introduced into the HLA-B moiety to improve stability and production. *In vitro*, IOS-1002 was demonstrated to bind LILRB1 with nanomolar affinities and to react also with LILRB2 as well as KIR3DL1. The fusion protein blocked the interaction between HLA-G and both LILRB1 and LILRB2, thereby impairing ITIM signaling by reducing phosphorylation of SHP1 and SHP2 adapter molecules. IOS-1002 shifted macrophage polarization towards an inflammatory M1 phenotype, increased tumor cell phagocytosis *in vitro* as single agent and enhanced cytotoxicity by T and NK cells against tumor cells. Therapeutic efficacy was demonstrated *in vivo* in syngeneic murine models of colon cancer and in non-small cell lung cancer patient-derived xenografts in human immune system mice. A phase 1a/1b study to evaluate IOS-1002 in adult patients with advanced solid tumors has received approval (NCT05763004) recently. IOS-1002 will be investigated as monotherapy or in combination with a PD-1 antibody.

## Concluding remarks

Pre-clinical investigations have identified the HLA class I:LILRB1 axis as an attractive target for immune checkpoint inhibition, as its blockade may allow to promote both adaptive and innate immune responses. Promising results were obtained with combinations of LILRB1-directed antibodies and tumor targeting antibodies, bispecific CD3 antibodies or other immune checkpoint inhibitors blocking CD47 or PD-1. However, the results were obtained with individual anti-LILRB1 antibodies, combination partners or in models of distinct tumor entities, and further studies are required to draw general conclusions. Interestingly, in different studies individual LILRB1 antibodies differed in their activity to induce phagocytosis of tumor cells as single agent, which may be due to characteristics of target cells or of the antibody such as the isotype or the epitope specificity. Yet, direct comparisons between antibodies are missing, and the ideal format for LILRB1 blocking agents needs to be determined.

Moreover, it is not clear if or to what extent the expression of other inhibitory receptors may impact the efficacy of LILRB1 blockade. Thus, other ‘Don´t Eat Me!’ signals beside HLA class I and CD47, such as PD-L1, CD24, adipocyte plasma membrane-associated protein (APMAP) or signaling lymphocyte activation molecule (SLAM) (9, 145–147), may hamper phagocytosis and diminish the effects by LILRB1 blockade. In addition, the presence of antigens with pro-phagocytotic function such as the ‘Eat Me!’ signal molecule calreticulin may play a role (148). Moreover, knowledge on the interplay between LILRB1 with other inhibitory receptors recognizing HLA or other cellular ligands in NK cells and T cells is still patchy and requires further investigation.

The encouraging preclinical results laid the foundation to evaluate individual LILRB1 antibodies clinically. Yet, current clinical studies focus on solid tumors. LILRB1 blockade may be also effective against leukemias and lymphomas. These may more likely express LILRB1, which needs to be taken into consideration. Potential pitfalls in the clinical application of LILRB1 directed antibodies may arise from antigen sink due to LILRB1 expression by tumor cells or immune cells that may not contribute to eradication or play only minor roles. Also, side effects of LILRB1 blockade may derive from its broad expression pattern including various types of leukocytes and osteoclast precursors. For example, genetic deletion of the murine LILRB ortholog PirB was shown to accelerate osteoclastogenesis in mice, though the development of osteoporosis was not observed in this study (58).

Beside LILRB1 also other LIR represent potential target antigens in immunotherapy. For instance, LILRB2, LILRB3 and LILRB4 were described as myeloid immune checkpoints and anti-LILRB2 and anti-LIRB4 antibodies have entered clinical trials recently (18, 149–155). It will be interesting to see, whether the pre-clinical success of targeting LILRB1 or other LIR can be translated into clinical application.

## Author contributions

TZ and CK conceived the article structure and prepared the initial draft of the manuscript. TZ prepared the table and designed figures. All authors contributed to writing, editing and proofreading the manuscript.
